# Cutaneous Angiosarcoma Mimicking Masson’s Tumor in an Elderly Male: A Diagnostic Challenge and Treatment Failure

**DOI:** 10.7759/cureus.93727

**Published:** 2025-10-02

**Authors:** Maryam Alzaabi, Ali Alroumi, Humoud Al-Sabah

**Affiliations:** 1 Dermatology, As'ad K. Al-Hamad Dermatological Center, Kuwait, KWT; 2 Dermatology, Faculty of Medicine, Kuwait University, Kuwait, KWT; 3 Dermatopathology, As'ad K. Al-Hamad Dermatological Center, Kuwait, KWT

**Keywords:** biopsy, cutaneous angiosarcoma, dermatopathology, facial lesion, histopathology, malignant vascular lesion, masson's tumor, vascular tumors

## Abstract

Cutaneous angiosarcoma is a rare tumor that may resemble other vascular lesions, such as Masson’s tumor. It may present as asymptomatic nodules, patches, or plaques, which are most commonly seen in the central face, scalp, or neck. It is an aggressive cancer that originates from endothelial cells that line blood or lymphatic vessels, and since it may present similarly to other benign tumors, recognizing it is essential to prevent the risk of further growth and potential metastasis. This case report presents an elderly male with an asymptomatic facial lesion, initially diagnosed as Masson’s tumor, but later recognized as cutaneous angiosarcoma. We present a previously healthy 82-year-old African male with a two-month history of an asymptomatic facial lesion. Examination revealed multifocal erythematous to violaceous nodular growths involving both cheeks, the nasal bridge, and the periocular regions. This case report emphasizes the importance of considering cutaneous angiosarcoma in elderly patients presenting with asymptomatic skin lesions.

## Introduction

Cutaneous angiosarcoma is a rare and aggressive soft tissue tumor that originates from endothelial cells of blood and lymphatic vessels. It is highly invasive and has a high metastatic potential, manifesting as a bruise-like erythematous nodule, patch, or plaque affecting the skin of the face, scalp, or neck. Because its presentation is nonspecific, it may easily be mistaken for other benign skin lesions. This case report revolves around an 82-year-old male with an asymptomatic facial lesion and aims to provide a detailed account of the patient’s clinical presentation, laboratory investigations, and histopathological findings.

## Case presentation

An 82-year-old African male presented with a progressively enlarging, indurated skin lesion on the mid-face, first noticed two months earlier. The lesion was asymptomatic, non-tender, and free from oozing or bleeding. One week prior, he had undergone excision of a presumed pyogenic granuloma on the nose in the otorhinolaryngology department. At that time, there was no evidence of spread beyond the primary site, and examination of the cervical, submental, and supraclavicular regions revealed no lymphadenopathy. The patient’s past medical history was notable for hypertension. He had no smoking history, no exposure to immunosuppressive drugs, and no family history of angiosarcoma. He worked indoors and otherwise enjoyed good health.

**Figure 1:**
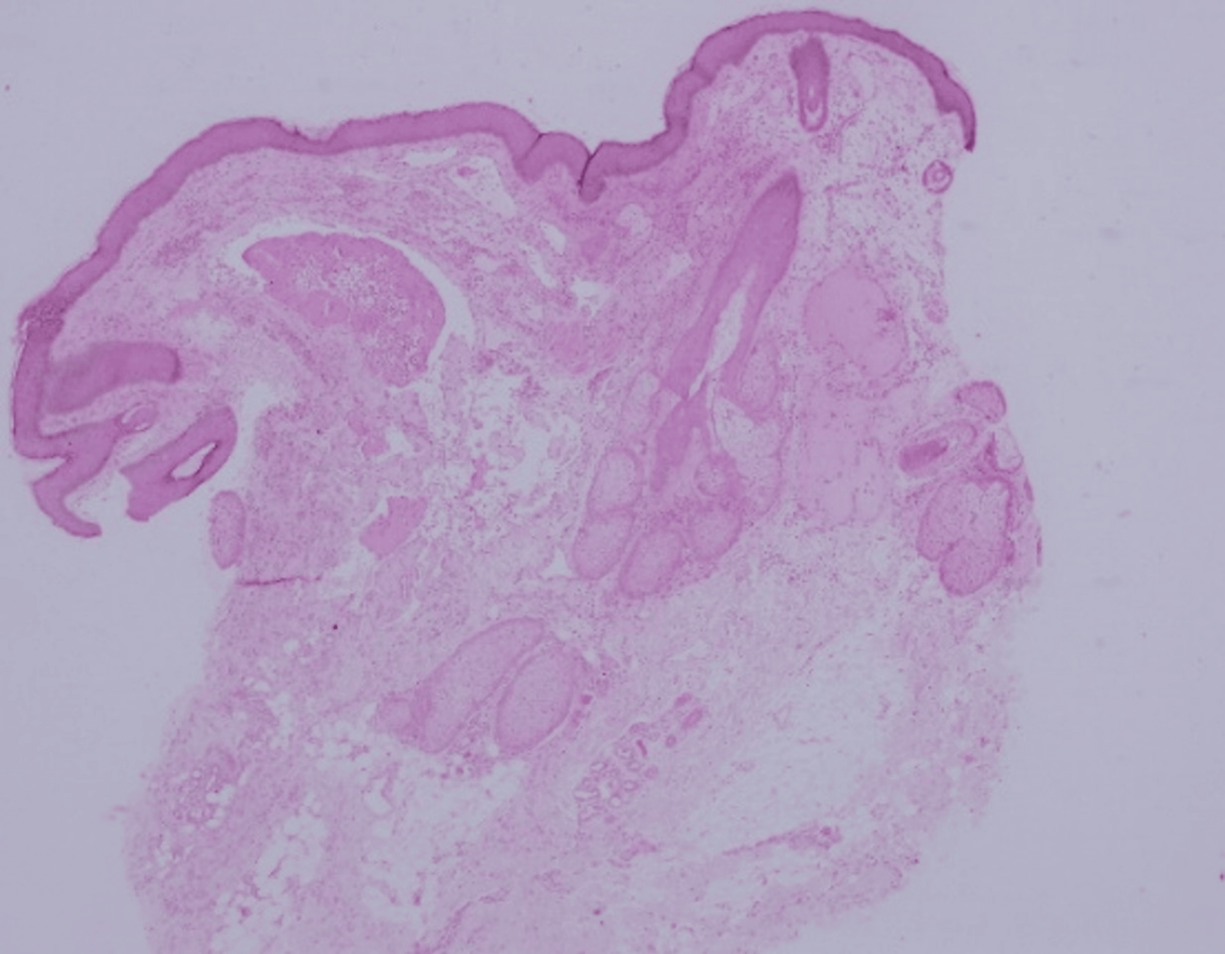
An initial biopsy from a nodule showed prominent papillary endothelial proliferation within a vascular space, with thickened hyperplastic endothelium forming non-encapsulated papillary structures infiltrating the stroma, consistent with Masson’s tumor (low magnification x4, with hematoxylin & eosin stain).

**Figure 2:**
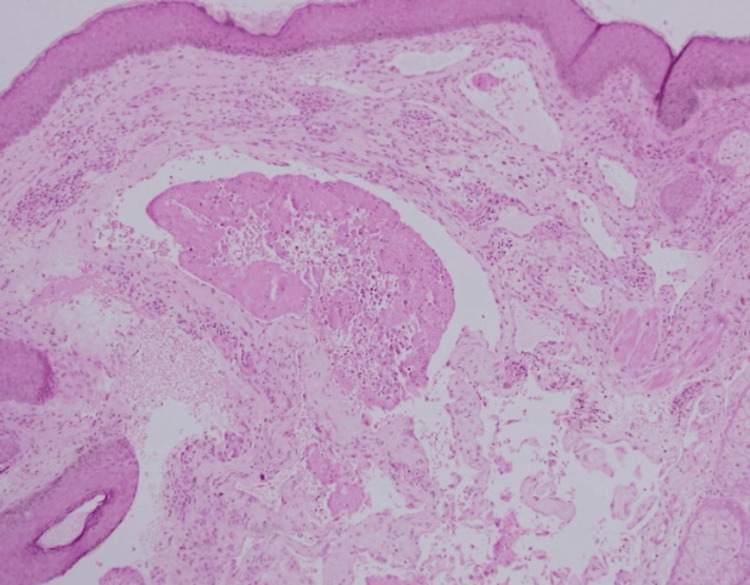
Under higher magnification, papillary structures were lined by flattened endothelial cells with mild atypia and no significant mitotic activity. Vascular channels contained red blood cells and minimal fibrin deposits, with early thrombosis noted in some areas (intermediate magnification x10, with hematoxylin & eosin stain).

**Figure 3:**
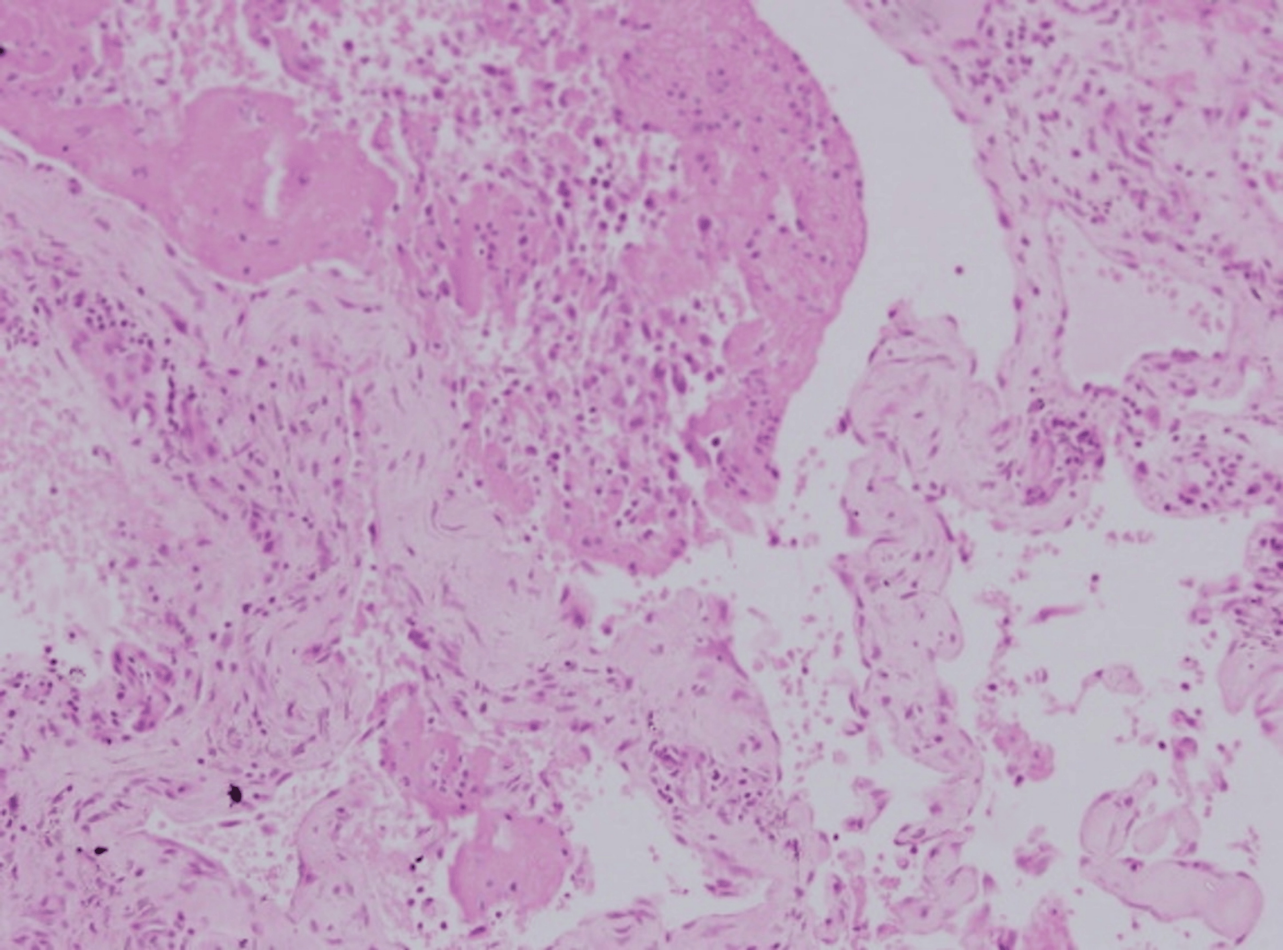
The lesion’s intravascular location showed multiple small, rounded papillae covered by hyperplastic endothelial cells, projecting into the lumen of an existing blood vessel, without destructive growth or significant atypia, supporting a benign nature (high magnification x40, with hematoxylin & eosin stain).

**Figure 4:**
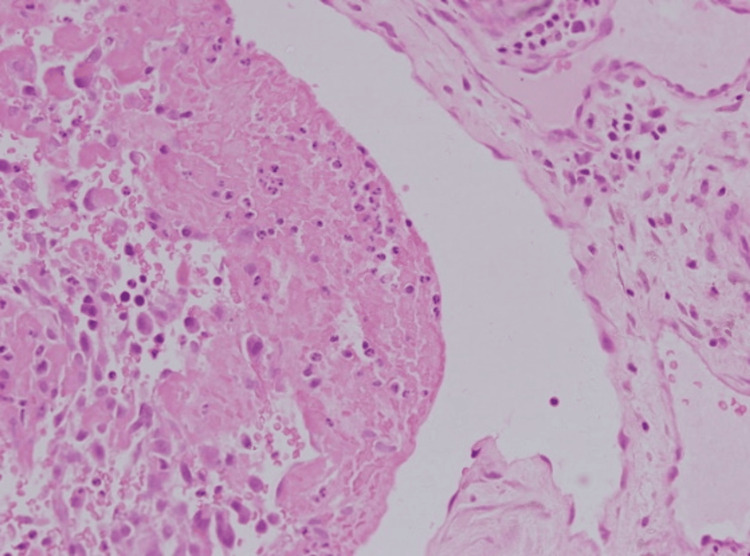
Several vascular channels lined by mildly reactive endothelial cells appeared partially occluded by organized thrombi undergoing recanalization—a hallmark of Masson’s tumor. The surrounding tissue had low cellularity and mild inflammation (high magnification x100, with hematoxylin & eosin stain).

**Figure 5:**
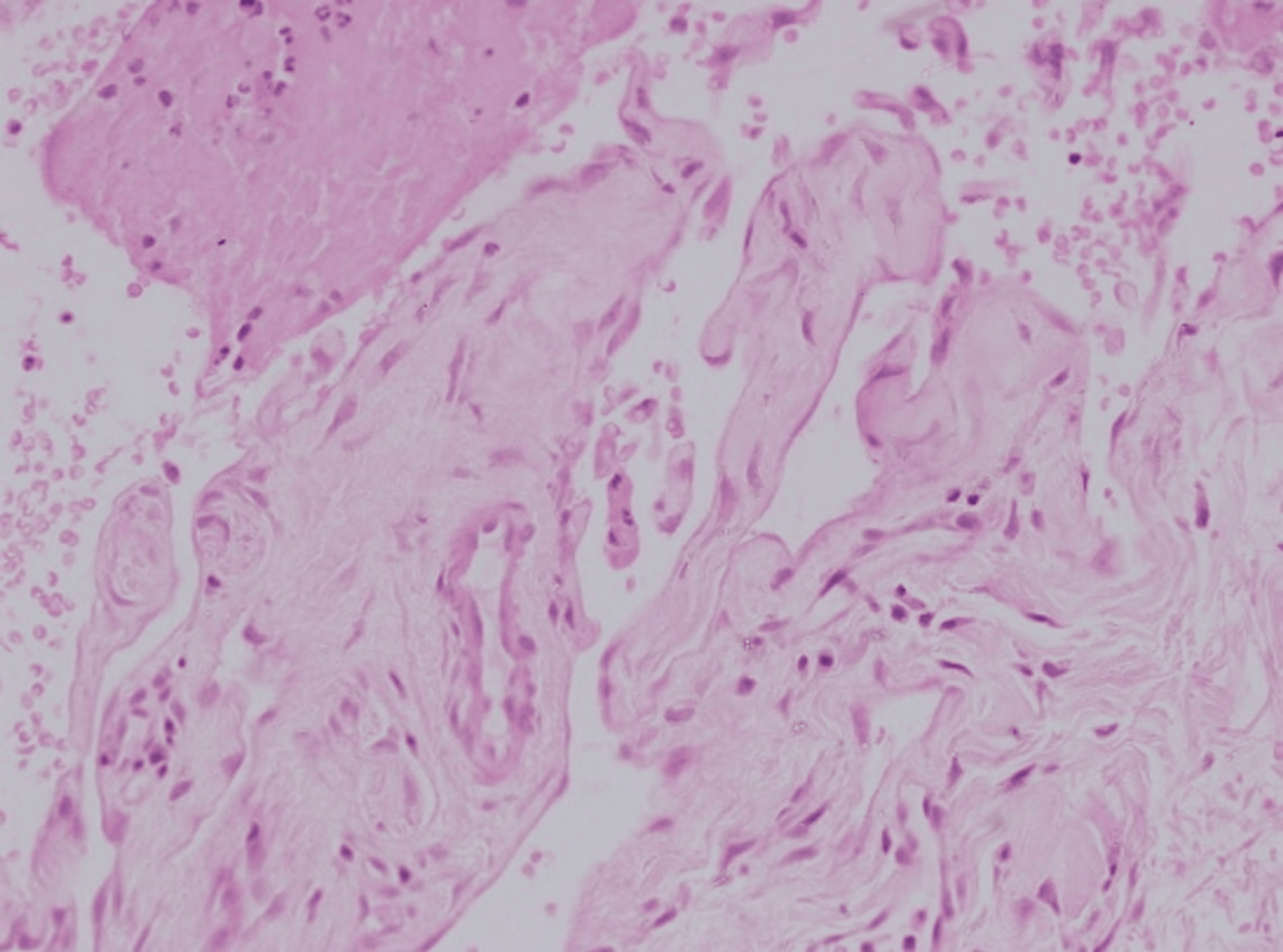
Extensive endothelial proliferation nearly occluded the vascular lumen, with endothelial cells showing a slightly increased nucleus-to-cytoplasm ratio but maintaining a benign appearance. Focal fibrin deposits and early thrombus organization were critical diagnostic features (high magnification x100, with hematoxylin & eosin stain).

An initial biopsy of the mid-face lesion suggested intravascular papillary endothelial hyperplasia (Masson’s tumor), a benign vascular lesion characterized by papillary endothelial proliferations within a vascular lumen (Figures 1-5). Given the atypical and progressively enlarging appearance, the patient was treated with three sessions of external beam radiation therapy over a six-week period.

**Figure 6:**
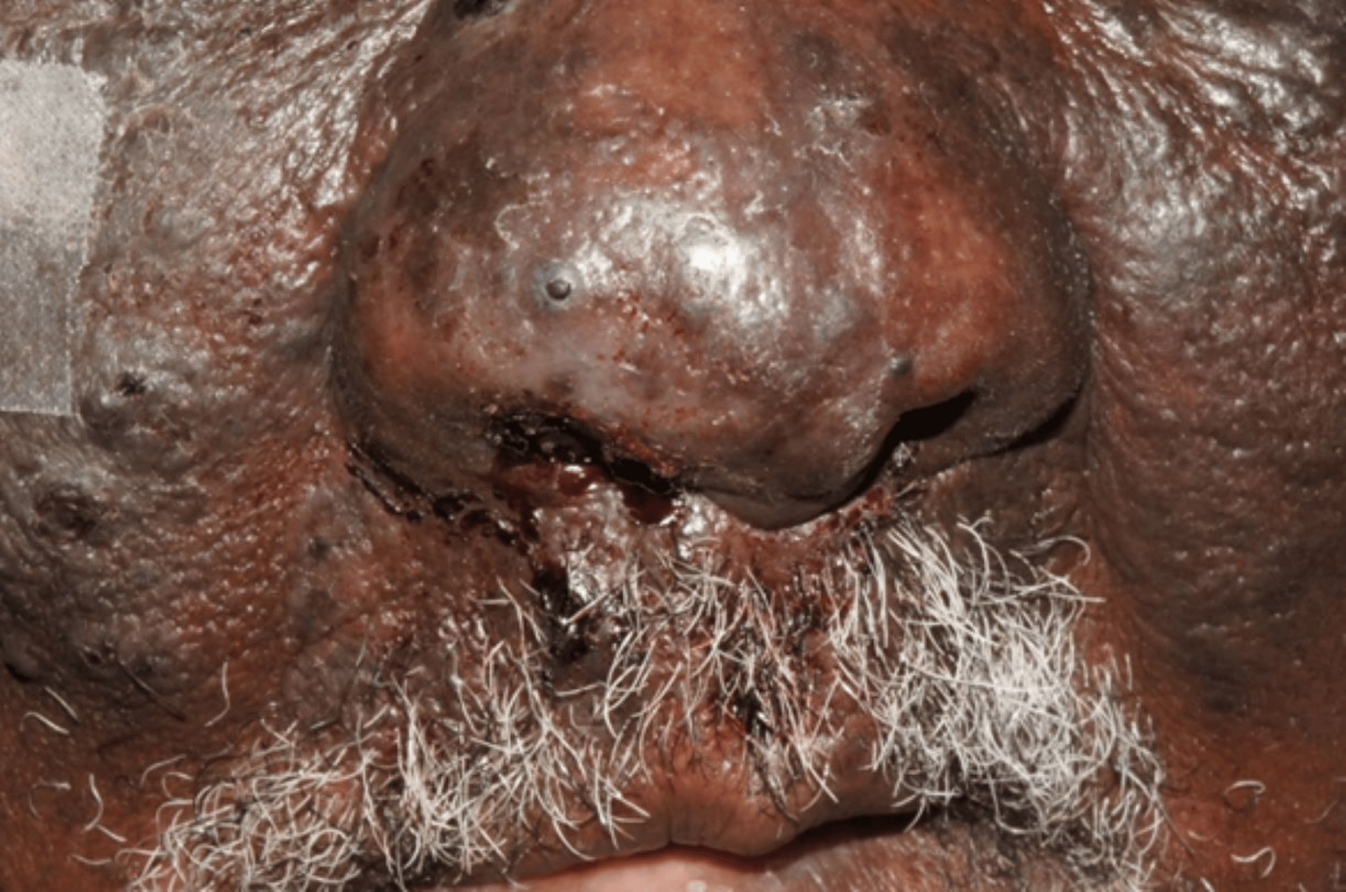
The facial lesion consists of multifocal, indurated nodules with a violaceous hue, primarily affecting the cheeks, nasal bridge, and periocular areas, with signs of superficial ulceration and crusting.

**Figure 7:**
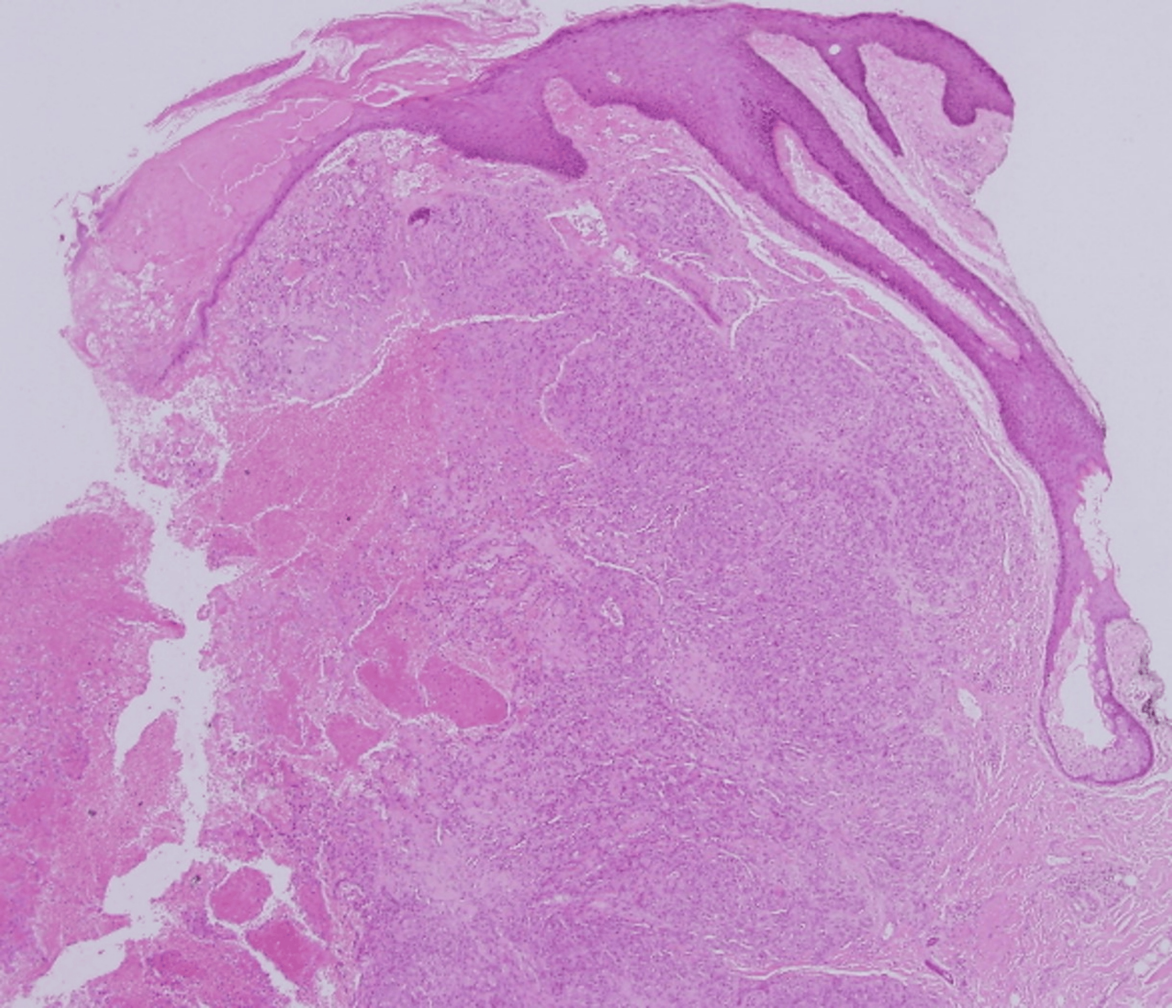
The second biopsy showed a highly cellular tumor infiltrating the dermal and subcutaneous layers, with irregular, blood-filled channels lined by atypical endothelial cells, indicating neovascularization. The cells displayed pleomorphism and hyperchromasia, with rudimentary lumen formation in some areas. Sparse inflammatory infiltrates were present in the reactive stroma (low magnification x4, with hematoxylin & eosin stain).

**Figure 8:**
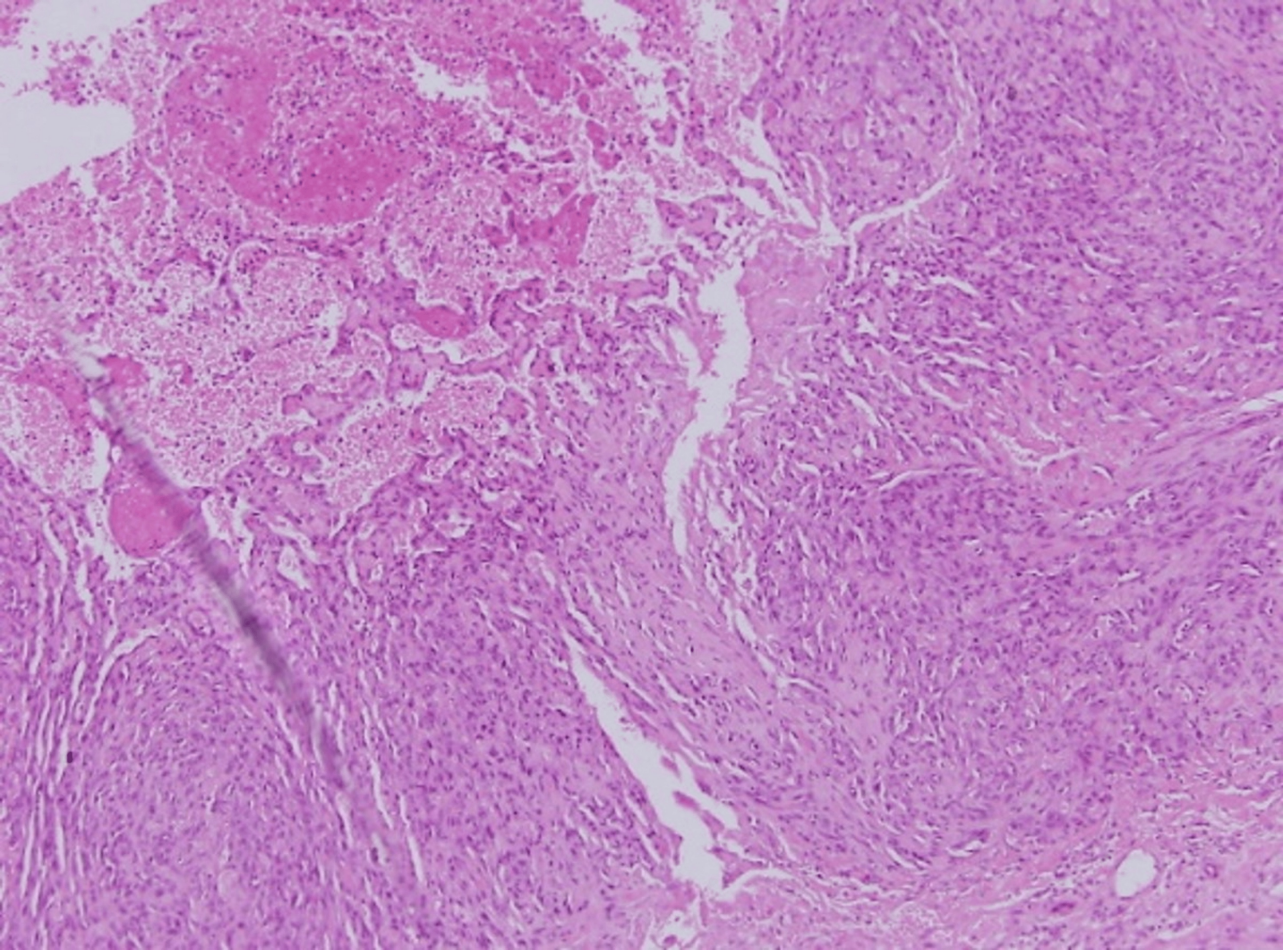
At higher magnification, the tumor’s aggressiveness was evident, with significant nuclear atypia, numerous mitotic figures, and disorganized vascular channels disrupting the collagenous stroma—indicating malignant invasion into adjacent tissues (intermediate magnification x10, with hematoxylin & eosin stain).

**Figure 9:**
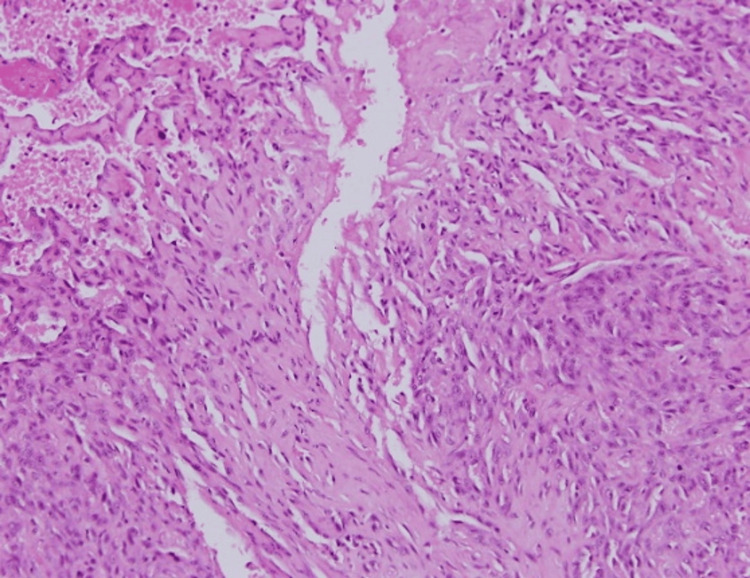
Spindle-shaped endothelial cells with irregular nuclei and visible nucleoli were embedded within a myxoid stroma, alongside sporadic multinucleated giant cells (high magnification x40, with hematoxylin & eosin stain).

**Figure 10:**
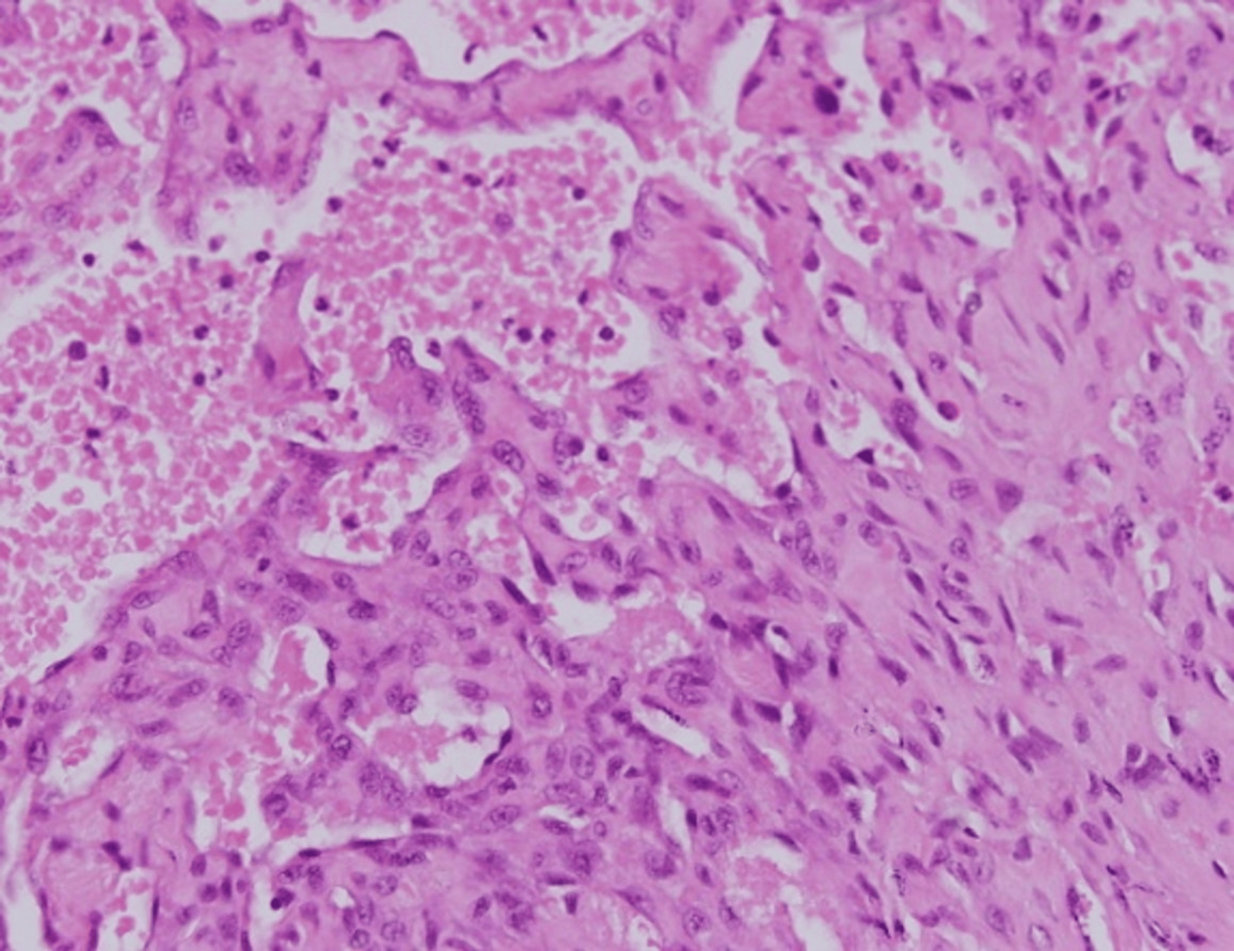
Higher magnification of Figure 9 showing spindle-shaped endothelial cells with irregular nuclei and prominent nucleoli within myxoid stroma, accompanied by occasional multinucleated giant cells. These features aligned more closely with cutaneous angiosarcoma than with Masson’s tumor (high magnification x100, with hematoxylin & eosin stain).

On re-examination following radiation, the patient exhibited extensive cutaneous lesions across the mid-face, progressing from a localized vascular mass to multifocal, nodular growths involving both cheeks, the nasal bridge, and periocular regions. The lesions appeared erythematous to violaceous, with areas of superficial ulceration, crusting, and thickened, indurated skin. Swelling in these regions suggested possible deeper tissue involvement (Figure 6). Although these clinical findings raised concern for an aggressive vascular malignancy such as angiosarcoma, they were not diagnostic and prompted urgent repeat biopsy for confirmation. Facial nerve function remained intact, and lymph node examination was unremarkable. Since the lesion continued to enlarge, a second biopsy was performed from the same site (Figures 7-10).

Laboratory investigations were within normal ranges (Table 1). Imaging with CT and MRI showed no evidence of metastatic disease, and echocardiography revealed no abnormalities. These findings supported the localization of the disease without systemic spread.

**Table 1:** Blood tests.

Blood tests	Result	Normal range
Complete blood count - white blood cells	6.6	4-10 x 10 ^9/L
Complete blood count - red blood cells	4.3	3.8-4.8 x 10^12/L
Platelets	203	150-410 x 10^9/L
Liver function test - alanine aminotransferase (ALT)	15	3-50 U/L
Liver function test - aspartate aminotransferase (AST)	22	3-50 U/L
Liver function test - gamma-glutamyl transferase (GGT)	11	5-42 U/L
Renal function test - creatinine	83	64-104 umol/L
Thyroid hormone	2.31	0.8-3.9 ulU/ml
Parathyroid hormone	5.7	1.3-9.3 pmol/L
Vitamin D	90	>75 nmol/L - sufficient; >250 nmol/L - critical high
Serum calcium	2.32	2.2-2.6 mmol/L
Alkaline phosphatase	103	52-171 U/L

Histopathological confirmation, together with the lesion’s clinical features, supported the final diagnosis of cutaneous angiosarcoma.

## Discussion

Cutaneous angiosarcoma is a condition characterized by aggressive vascular or lymphatic endothelial proliferation. It was first described in detail by Wilson Jones in 1964, and may rapidly evolve into larger ulcerative growths [1]. Because this type of soft tissue sarcoma initially presents indolently and with variable presentations, most cases are either misdiagnosed or diagnosed late, making prognosis relatively poor due to its high metastatic potential [2].

Angiosarcoma accounts for approximately 2% of all soft tissue sarcomas, and most appear spontaneously, although it is commonly seen following radiotherapy [1]. The condition typically affects older individuals, with the incidence peaking at around the seventh decade of life, but other subtypes may occur in younger patients. It has no sex predilection, but is most reported in elderly white males. Few studies have shown an association with BRCA1 and BRCA2 gene mutations, along with familial syndromes such as neurofibromatosis, Maffucci syndrome, or Klippel-Trenaunay syndrome. Environmental factors like chronic UV exposure and certain chemicals like vinyl chloride, arsenic, radium, or anabolic steroids may also trigger angiosarcoma growth [3]. Chronic lymphedema (Stewart-Treves syndrome) and prior radiotherapy are also recognized triggers. While the exact pathogenesis remains unclear, underlying triggers result in significant vascular or lymphatic endothelial cell proliferation, which is why angiosarcoma may be classified as either vascular, lymphatic, or mixed according to immunohistochemistry [4]. As such, abnormal, pleomorphic, and malignant endothelial cells must be present to diagnose the condition as cutaneous angiosarcoma.

Although the condition may occur anywhere, it is most commonly seen in the head and neck regions [5]. A thorough and detailed history may provide insight into any underlying cutaneous diseases or previous exposure to radiotherapy or certain triggers, and an extensive physical examination may help in determining the severity of the lesion. It typically presents as multifocal and bruise-like lesions consisting of purple-red nodules, patches, or plaques. With further growth, the lesion may present with bleeding and ulceration. Laboratory tests, such as complete blood count, biochemical profile, liver or renal function tests, and inflammatory markers, are usually unremarkable unless the tumor has spread viscerally and has affected other soft tissues. As such, imaging with CT, MRI, or PET may be essential in assessing the extent of tumor growth and spread. Biopsy of the lesion shows spindled, polygonal, epithelioid, and primitive round cells, with immunohistochemistry showing increased expression of various factors like factor VIII, CD31, CD34, ERG, FLI1, and VEGF [6].

Still, diagnosis may be challenging due to its resemblance to many vascular and benign tumors, one of them being Masson’s tumor. Also called intravascular papillary endothelial hyperplasia (IPEH), this condition and angiosarcoma are both vascular lesions but differ significantly in their clinical behavior and pathology. Masson’s tumor is a benign and relatively uncommon lesion characterized by endothelial cell proliferation within blood vessels, often in association with thrombosis [7]. It typically presents as a solitary and painless nodular lesion that grows slowly. This lesion can occur at any age but most commonly in adults, and shows no significant sex predilection. It is typically found in the skin and subcutaneous tissues, especially in the head, neck, and extremities, but can also appear in unusual locations such as the oral cavity, liver, or central nervous system. When having both diseases as differential diagnoses, histopathological and immunohistochemical analyses are essential in differentiating between them, since clinical features alone can be misleading. Small or non-representative biopsies may also contribute to misdiagnosis, necessitating repeat sampling if suspicion persists. Most cases of cutaneous angiosarcoma occur spontaneously or post radiotherapy, whereas most cases of IPEH occur following recent trauma. Histologically, while Masson’s tumor shows localized proliferation of endothelial cells within a thrombosed vessel, angiosarcoma exhibits uncontrolled growth of endothelial cells, with a high risk of spreading to other organs. Cutaneous angiosarcoma is characterized by aggressive, atypical endothelial cell proliferation forming disorganized and infiltrative vascular channels, with notable pleomorphism and high mitotic activity, indicative of its malignant nature and potential for rapid progression [8].

The primary clinical mimics of angiosarcoma include rosacea, localized bruising, Kaposi sarcoma, amelanotic melanoma, hemangioma, dermatofibrosarcoma protuberans (DFSP), pyogenic granuloma, squamous cell carcinoma, and cutaneous lymphoma. Differentiating angiosarcoma from these conditions relies on a combination of clinical features, histopathological analysis, and immunohistochemical staining. For example, Kaposi sarcoma, which presents with similar vascular lesions, can be distinguished by its association with human herpesvirus-8 (HHV-8), detected by immunohistochemistry, and characteristic spindle cell proliferation. Amelanotic melanoma, another potential mimic, is identifiable by markers such as S-100 and HMB-45, which are absent in angiosarcoma. Hemangiomas generally display a more organized structure and appear earlier in life, unlike the infiltrative growth pattern typical of angiosarcoma. DFSP, distinguished by a storiform pattern of spindle cells, also stains positive for CD34, aiding in its identification [9]. Pyogenic granuloma, a benign vascular proliferation, can often be excluded based on its rapid growth following trauma and the absence of atypical or mitotically active cells. Squamous cell carcinoma typically presents with keratin pearls and markers like p63, which contrast with the atypical vascular channels characteristic of angiosarcoma. Finally, cutaneous lymphomas can be ruled out through their unique immunophenotypic profiles, distinct from the endothelial markers expressed in angiosarcoma [10]. Clarifying which are immunohistochemical endothelial markers (CD31, ERG, FLI1) versus epithelial or viral stains (p63, keratin pearls, HHV-8) avoids conflating distinct methodologies. Clinically, multiple biopsies are recommended for proper diagnosis, as a single biopsy might not be sufficient in diagnosing angiosarcoma, especially if the lesions are large or multifocal.

Radiation-induced angiosarcoma has been reported, often associated with c-Myc amplification. In our case, radiation was administered after the initial biopsy suggested a Masson’s tumor. While it is possible that treatment contributed to disease progression, it is more likely that the first biopsy was non-representative and the lesion was angiosarcoma from the outset [10].

While there is still no definitive and ideal treatment for cutaneous angiosarcoma, surgical excision remains the most widely accepted option, with presurgical and postsurgical radiotherapy. Nonetheless, the result may be cosmetically unpleasing. In cases where the patient refuses to undergo surgery or if the lesion is unresectable, chemotherapy with paclitaxel may be considered [2]. For tumors resistant to chemotherapy, targeted therapy or immunotherapy may be considered. Ultimately, early diagnosis is essential in managing the patient’s condition, which is why it is important to consider cutaneous angiosarcoma even when dealing with an asymptomatic lesion that initially appears to be benign.

## Conclusions

In conclusion, this case highlights the diagnostic challenges posed by cutaneous angiosarcoma, particularly when it mimics benign vascular lesions like Masson’s tumor. The initial misdiagnosis underscores the importance of a high index of suspicion and the need for repeated biopsies in cases where lesions exhibit atypical growth or fail to respond to standard treatment. Early and accurate identification of angiosarcoma is crucial, given its aggressive nature and potential for metastasis. This case reinforces the value of thorough histopathological evaluation and the role of clinicians in considering angiosarcoma as a differential diagnosis in elderly patients presenting with persistent, asymptomatic vascular lesions.

## References

[1] Jones EW (1964). Malignant angioendothelioma of the skin. Br J Dermatol.

[2] Guan L, Palmeri M, Groisberg R (2023). Cutaneous angiosarcoma: a review of current evidence for treatment with checkpoint inhibitors. Front Med (Lausanne).

[3] Spiker AM, Mangla A, Ramsey ML (2025). Angiosarcoma. StatPearls.

[4] Kamo R, Ishii M (2011). Histological differentiation, histogenesis and prognosis of cutaneous angiosarcoma. Osaka City Med J.

[5] Albores-Saavedra J, Schwartz AM, Henson DE, Kostun L, Hart A, Angeles-Albores D, Chablé-Montero F (2011). Cutaneous angiosarcoma. Analysis of 434 cases from the Surveillance, Epidemiology, and End Results Program, 1973-2007. Ann Diagn Pathol.

[6] Cao J, Wang J, He C, Fang M (2019). Angiosarcoma: a review of diagnosis and current treatment. Am J Cancer Res.

[7] Sasso SE, Naspolini AP, Milanez TB, Suchard G (2019). Masson's tumor (intravascular papillary endothelial hyperplasia). An Bras Dermatol.

[8] Lee KB, Lee HS, Lee HE (2006). Immunohistochemical characteristics of Kaposi sarcoma and its mimicries. J Pathol Transl Med.

[9] Alkabbaa S, Alsaedan AM, Alammar AK (2023). Pyogenic granuloma of the thumb mimicking squamous cell carcinoma of the nail subunit: case report and review of the literature. J Surg Case Rep.

[10] Gupta A, Chaturvedi S, Jha K, Nazir W (2017). Intravascular papillary endothelial hyperplasia presenting as a cystic mass in the scalp with underlying bone involvement: a rare entity. Int J Appl Basic Med Res.

